# Depression and anxiety in inflammatory bowel disease: mechanisms and emerging therapeutics targeting the microbiota-gut-brain axis

**DOI:** 10.3389/fimmu.2025.1676160

**Published:** 2025-11-07

**Authors:** Yan Qian, Yang Chen, Linquan Liu, Tiesong Wu, Xiehui Chen, Guiping Ma

**Affiliations:** 1 Public Relations Section, Shenzhen Longhua District Central Hospital, Shenzhen, China; 2 Department of Emergency, Shenzhen Longhua District Central Hospital, Shenzhen, China; 3 Chronic Disease Management Department, The First Hospital of Hunan University of Chinese Medicine, Changsha, China; 4 Department of Pharmacy, Shenzhen Longhua District Central Hospital, Affiliated Longhua Hospital of Shenzhen University, Shenzhen, China; 5 Department of Geriatric Medicine, Shenzhen Longhua District Central Hospital, Shenzhen, China; 6 Beijing University of Chinese Medicine Affiliated Shenzhen Hospital (Longgang), Shenzhen, China

**Keywords:** depression and anxiety, gut microbiome, inflammatory bowel disease, prevention and treatment, public health management

## Abstract

Inflammatory bowel disease (IBD), encompassing ulcerative colitis (UC) and Crohn’s disease (CD), represents a group of chronic, relapsing intestinal inflammatory disorders with incompletely understood etiology. Depression and anxiety, as prevalent psychiatric conditions, exhibit rising incidence rates; notably, IBD patients demonstrate heightened susceptibility to these disorders compared to the general population, thereby exacerbating disease burden and increasing risks of adverse clinical outcomes. Emerging evidence reveals shared pathophysiological mechanisms between IBD and depression/anxiety. This review specifically addresses depression and anxiety within the IBD disease context, integrating recent epidemiological evidence and risk factors. Centered on the gut-brain axis framework, we examine mechanistic underpinnings through two interconnected pathways: gut dysbiosis and neuroimmune interactions mediated by inflammatory cytokines and neurotransmitters. Finally, we explore therapeutic interventions for depression and anxiety in IBD based on these mechanistic insights, aiming to advance clinical and public health management strategies.

## 1 Introduction

Inflammatory Bowel Disease (IBD) is a group of chronic, relapsing inflammatory bowel diseases whose etiology has not been fully clarified; it primarily includes Ulcerative Colitis (UC) and Crohn’s Disease (CD) (1). The characteristic pathological changes of IBD involve impaired intestinal mucosal barrier function, abnormal activation of the immune system, and persistent inflammatory responses, ultimately leading to disturbances in intestinal structure and function (2, 3). UC is characterized by continuous superficial inflammation that primarily involves the large intestine (colon), typically originating from the rectum and extending proximally in a contiguous pattern (4). In contrast, Crohn’s disease (CD) can affect any segment of the gastrointestinal tract, most commonly presenting as a patchy distribution with preferential involvement of the small intestine, particularly the terminal ileum. In some cases, distinguishing between CD and UC may be challenging, resulting in an interim diagnosis of “indeterminate” or “unclassified” colitis and potential delays in treatment (5, 6).

Over the past decade, the incidence and prevalence of IBD have been on an upward trend (7, 8). IBD patients are more susceptible to mental disorders, particularly anxiety and depression, compared to the general population, and these comorbidities exacerbate the disease burden by increasing healthcare resource utilization, hospitalization risks, and readmission rates (9, 10). The bidirectional comorbidity mechanisms between IBD and anxiety/depression are complex, involving genetic correlations between IBD and anxiety/depression, induction of anxiety and depression through hormonal and inflammatory signaling pathways, dysregulation of the gut-microbiota-brain axis, and gut-immune-brain axis cascades—such as systemic inflammation triggered by chronic bowel inflammation breaching the intestinal barrier, which transmits signals to the central nervous system (11, 12). However, mental health issues in IBD, including depression and anxiety symptoms, have become a global public health concern, urgently demanding the development of prevention and management strategies for mental disorders in IBD (13, 14). Therefore, deeply understanding the mechanisms underlying depression and anxiety in IBD based on the gut-microbiota-brain axis theory can help provide comprehensive mental health guidance for primary healthcare policymakers and formulate more holistic and effective diagnostic and therapeutic strategies to improve patients’ overall health.

This study aims to review existing research, analyze the prevalence and risk factors of depression and anxiety in IBD, and explore the mechanisms of their occurrence in IBD patients from two aspects: gut microbiota dysbiosis and neuroimmune interactions mediated by inflammatory cytokines and neurotransmitters, thereby offering new insights and directions for mental health prevention and management in the IBD population.

## 2 Depression and anxiety in IBD: prevalence and bidirectional association

Depression and anxiety are common comorbidities in IBD. Recent meta-analyses reveal that among 300 IBD participants, 39.0% reported symptoms of common mental disorders, with 35.7% exhibiting anxiety and 15.7% depression (15). Younger age, female sex, tobacco use, longer duration of pre-diagnostic symptoms, higher gastrointestinal symptom-specific anxiety, and stressful life events within the past 12 months were significantly associated with increased likelihood of these psychiatric symptoms (15, 16). During over 150,000 person-years of follow-up, IBD patients demonstrated elevated risks for anxiety (OR 1.4; 95% CI 1.2–1.7) and depression (OR 1.4; 95% CI 1.3–1.6), commencing at least five years before IBD diagnosis and persisting for at least a decade post-diagnosis (anxiety HR 1.3; 95% CI 1.1–1.5; depression HR 1.5; 95% CI 1.4–1.7) (17). A study of 48,799 newly diagnosed IBD cases indicated significantly higher psychiatric incidence versus healthy controls: anxiety IRR 1.17 (1.11–1.24) and depression IRR 1.36 (1.31–1.42) (18). CD patients showed particularly pronounced risks: anxiety HR 1.38 (1.16–1.65) and depression HR 1.36 (1.26–1.47), with peak mental disorder risk occurring within the first year post-IBD diagnosis (18). These findings robustly support the high prevalence of depression and anxiety in IBD.

Studies indicate a complex bidirectional association between depression and IBD. On one hand, individuals with depression exhibit a significantly elevated risk of developing IBD (19–21), and depression exacerbates clinical symptoms in IBD patients, increasing flare-ups, rehospitalizations, and surgical risks (22). Conversely, antidepressant therapy demonstrates selective protective effects against IBD, with differential efficacy across antidepressant classes for CD and UC (20). Genetic research further elucidates potential mechanisms: genome-wide association studies confirm a causal effect of depression on IBD (23), while evidence for reverse causality remains weaker (24). These results suggest that although genetic factors play a crucial role in depression-mediated IBD pathogenesis, the precise mechanisms and objective influencing factors underlying anxiety/depression development in IBD patients require further clarification. In-depth elucidation of these pathophysiological mechanisms will provide novel intervention targets and therapeutic strategies for comprehensive management of psychological comorbidities in IBD.

## 3 Mechanisms underlying depression and anxiety in IBD

The chronic disease burden and uncertainty in IBD patients may lead to psychological stress, where maladaptive coping mechanisms can increase vulnerability to depression (25). Patients’ perceptions and understanding of their illness—termed illness cognition—also impact mental health, with negative illness perceptions correlating with reduced quality of life and elevated depression levels (26). Personality traits (e.g., neuroticism) and external psychosocial stressors (e.g., pandemics) may modulate mental health outcomes in IBD patients (27). For instance, the COVID-19 pandemic significantly exacerbated psychological distress in this population (28, 29). During active disease phases, IBD patients exhibit higher depression prevalence than those in remission (30). Analyses from the Swiss IBD Cohort Study (SIBDCS) confirm that depressive symptoms strongly correlate with intestinal inflammatory activity and serve as critical predictors of clinical deterioration (31). A 12-month longitudinal study revealed that depressed IBD patients experienced significantly higher rates of: Disease flare-ups, Glucocorticoid usage, Treatment escalation, Hospitalizations or intestinal resections (32). Numerous studies demonstrate escalating depression and anxiety incidence with worsening IBD severity (33). Consequently, IBD patients face heightened susceptibility to these psychiatric comorbidities, mediated by the aforementioned psychological shifts and disease activity factors.

From a pathophysiological perspective, the development of depression and anxiety in IBD is governed by structural and molecular mechanisms (34). Comprehensive understanding of these biological substrates—including neuroimmune interactions, gut-brain axis dysregulation, and inflammatory cascades—is essential for developing targeted pharmacological interventions and precision management strategies.

### 3.1 Brain structural changes

The dextran sulfate sodium (DSS) and 2,4,6-trinitrobenzene sulfonic acid (TNBS) models exhibit distinct pathophysiological profiles (35): DSS induces Th2-mediated UC-like inflammation with superficial mucosal damage through direct epithelial toxicity (36), whereas TNBS triggers Th1-driven CD-like pathology characterized by transmural inflammation and granuloma formation (37). These divergent peripheral inflammatory patterns are mirrored in the central nervous system, with the prefrontal cortex (PFC) emerging as a critical mediator of IBD-associated neuropsychiatric symptoms (38). Comparative studies reveal model-specific mPFC remodeling: DSS exposure leads to reduced microglial immunoreactivity (Iba1/CD68 downregulation) and myelin protein depletion with concomitant Ranvier node disorganization, while TNBS challenge induces P2Y12 receptor upregulation and microglial hyperactivation (38, 39). Clinically, UC patients demonstrate mPFC hyperstability correlating with depression severity, alongside γ-aminobutyric acid (GABA)/Glx (a combination of glutamate and glutamine) metabolic deficits inversely linked to depressive symptoms (40). These findings collectively implicate mPFC dysfunction in IBD neuropsychiatric comorbidities through microglial priming and myelin disruption (38, 41).

**Figure 1:**
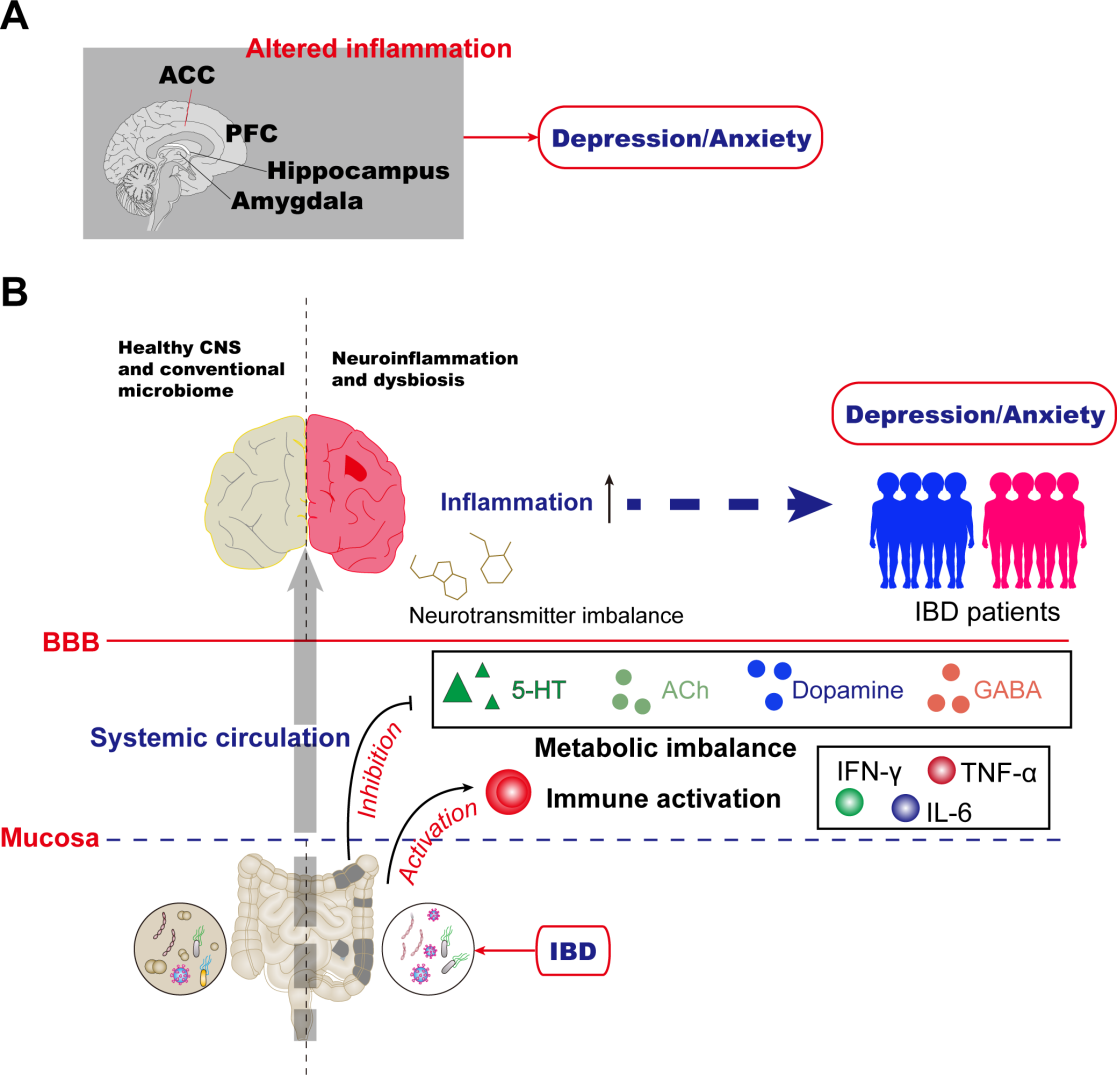
Mechanisms for anxiety and depression in inflammatory bowel disease. **(A)** Altered inflammation in the PFC, anterior cingulate cortex (ACC), amygdala, hippocampus, and other brain areas has been reported in humans. **(B)** The gut microbiome is essential for host immune functions and actively regulates mental health through microbiota-derived components and metabolites such as indoxyl sulfate, ACh, and norepinephrine (NE). In IBD patients, aberrations in gut microbiota homeostasis, or dysbiosis, are a common comorbidity that can lead to changes in metabolic imbalance and immune activation, resulting in altered neurotransmitter, and neuroinflammation. Collectively, these changes orchestrate the depression/anxiety observed in gut-brain interactions during IBD disease.

Neuroimaging studies uncover widespread brain structural remodeling characteristics in IBD patients (42). Compared to healthy controls, patients exhibit reduced gray matter volume (GMV) in multiple emotion-related brain regions, including the insula, thalamus, anterior knee of the cingulate gyrus, hippocampal complex, amygdala, and temporal pole, with more pronounced changes in active-stage patients (43, 44). Notably, disease duration negatively correlates with GMV in several brain regions (43). These findings suggest that, beyond the prefrontal cortex, structural alterations in the limbic system (e.g., hippocampus, amygdala) and cingulate gyrus collectively form the neurobiological basis for comorbid anxiety and depression in IBD patients (**Figure 1A**). These insights provide crucial clues for understanding the mechanisms of IBD and psychiatric symptom comorbidities.

### 3.2 Increased blood-brain barrier permeability

The BBB serves as a critical regulator of neuroimmune interactions, constituting a dynamic interface composed of brain microvascular endothelial cells, pericytes, neurons, astrocytes, and extracellular matrix (12, 45). Endothelial cells restrict paracellular diffusion of water-soluble substances by expressing tight junction proteins, solute carriers, and receptors, while facilitating selective transport of nutrients and metabolites from blood to the brain (46). Under physiological conditions, the BBB functions as an essential physical barrier limiting interactions between the peripheral immune system and the central nervous system (CNS) (47). In pathological states involving BBB dysfunction, increased permeability may exacerbate neuroinflammatory responses through immune cell infiltration and proinflammatory signaling (48).

Elevated levels of inflammatory cytokines—such as tumor necrosis factor-α (TNF-α), interleukin-1β (IL-1β), and interleukin-6 (IL-6)—in IBD patients can cross the BBB to directly affect neurotransmitter synthesis, release, and metabolism, thereby disrupting emotional regulation (49, 50). The chronic inflammatory state in IBD patients promotes the translocation of peripheral proinflammatory cytokines (TNF-α, IL-1β, IL-6) across the BBB, activating microglia and astrocytes in the brain (51). This leads to neuroinflammation and impairs synaptic plasticity (particularly in the hippocampus, a region crucial for emotional regulation), further aggravating depressive symptoms.

### 3.3 Alterations in gut microbiota and metabolites

Gut microbiota play a pivotal role in regulating the gut-brain axis (52, 53). IBD patients typically exhibit dysbiosis characterized by reduced microbial diversity, decreased beneficial bacteria, and increased harmful bacteria (54). Activation of microglia by gut-derived inflammatory signals could contribute to emotional dysregulation, a pathway supported by preclinical models of neuroinflammation (55, 56). Specific alterations (e.g., *Bacteroides, Faecalibacterium prausnitzii, Prevotella* spp. reduction (57) or *Proteobacteria* (58, 59), *Campylobacter concisus* (60) increase) promote the release of pro-inflammatory cytokines (IL-1β, IL-6, and TNF-α) (61–63), activating microglia (immune cells in the brain) and thereby disrupting emotional regulation (12, 64–66). A meta-analysis of 16S rRNA sequencing data from 1,200 IBD patients, which explicitly demonstrates dysbiosis patterns including reduced microbial diversity and depletion of beneficial genera (e.g., *Faecalibacterium*) in human IBD cohorts (54). *Roseburia* genus might harbor protective function against CD onset (57) and established IBD (67).

The gut microbiota plays a pivotal role in synthesizing neurotransmitters essential for mood regulation, such as serotonin (5-HT), acetylcholine (ACh), dopamine (DA), and gamma-aminobutyric acid (GABA) (68–70). Microbial dysbiosis can significantly impair neurotransmitter production or disrupt their metabolic pathways (71–73), thereby directly altering emotional states (**Figure 1B**). Notably, 5-HT secreted by enteroendocrine cells engages in bidirectional communication with specific gut bacteria, including *Turicibacter sanguinis*, which possesses serotonin uptake mechanisms critical for both microbial colonization and host physiological function (71). Beyond direct neurotransmitter synthesis, the gut microbiota modulates mood through the tryptophan metabolic pathway (74). As the primary precursor for 5-HT production, tryptophan metabolism is particularly vulnerable to microbial imbalance. Dysbiosis not only diminishes 5-HT synthesis but also promotes the accumulation of neurotoxic metabolites such as kynurenine (75). This phenomenon is clinically evident in IBD patients, where intestinal inflammation triggers excessive activation of the kynurenine (KYN) pathway, leading to elevated neurotoxic metabolites that significantly contribute to depressive symptomatology (76–78).

Short-chain fatty acids (SCFAs)—produced by microbial fermentation of dietary fiber—exert multifaceted benefits, including immune modulation and maintenance of gut barrier integrity (79). Reduced SCFA production in IBD patients compromises gut and brain health (80). Notably, butyrate and propionate possess anti-inflammatory properties and regulate microglial function; their deficiency potentiates neuroinflammation (81).

### 3.4 Intestinal inflammatory responses

The neural regulation of the gastrointestinal tract involves a complex multi-tiered nervous system (82). The gut is innervated by the CNS, autonomic nervous system (ANS), and enteric nervous system (ENS) (83, 84). The ENS—comprising the myenteric plexus and submucosal plexus—exhibits autonomous regulatory capacity and operates with relative independence from the CNS (85). The vagus nerve, a key component of the parasympathetic nervous system, plays a pivotal role in gastrointestinal modulation (86).

During IBD pathogenesis, intestinal inflammation activates the ENS (87). Inflammatory signals are transmitted to the brain via the vagus nerve, directly disrupting the synthesis and release of neurotransmitters (e.g., serotonin, dopamine) (88). These signals first reach the nucleus tractus solitarius (NTS) in the brainstem, then propagate to emotional regulatory centers such as the hypothalamus and amygdala (89). Chronic inflammatory stimulation amplifies pro-inflammatory signaling, impairing emotional regulation and inducing or exacerbating depression-like behaviors (90).

Increased intestinal permeability in IBD patients facilitates the translocation of lipopolysaccharide (LPS, an endotoxin from Gram-negative bacteria) into the bloodstream (50). This activates the peripheral immune system, triggering the release of pro-inflammatory cytokines (TNF-α, IL-6). These cytokines bind to receptors expressed on vagal afferents, altering central neurotransmitter release and behavior (33, 91–93). Studies indicate that administering pro-inflammatory cytokines to healthy volunteers or animals induces depression through this mechanism. Conversely, cytokine antagonists—such as anti-TNF therapy in IBD—alleviate disease-associated anxiety and depressive behaviors (94).

### 3.5 Impaired anti-inflammatory function of the vagus nerve

As a core conduit of the gut-brain axis, the vagus nerve exerts bidirectional regulatory effects in IBD-depression comorbidity (95). Its afferent fibers transduce intestinal inflammatory signals into neuroelectrochemical signals transmitted to the CNS, while its efferent fibers activate the cholinergic anti-inflammatory pathway (CAP) (96). CAP mediates the release of ACh, which binds to the α7 nicotinic acetylcholine receptor (α7nAChR) on immune cells, potently suppressing the release of pro-inflammatory cytokines such as TNF-α and establishing a neuro-immune negative feedback loop (97).

Clinical studies reveal that IBD patients commonly exhibit reduced vagal tone, impairing this anti-inflammatory function (98). This deficiency not only exacerbates intestinal inflammation but also induces depressive symptoms by disrupting hippocampal neuroplasticity (99).

### 3.6 HPA axis dysregulation

The hypothalamic-pituitary-adrenal (HPA) axis is a complex neuroendocrine system that maintains cortisol (CORT) homeostasis through negative feedback mechanisms, where cortisol inhibits hypothalamic and pituitary activity to reduce adrenocorticotropic hormone (ACTH) secretion, thereby regulating its own synthesis and release (100). This balanced system enables the body to adapt to various internal and external environmental changes, ensuring physiological stability. In DSS-induced colitis mice, HPA axis activation was observed to enhance pathogen clearance during the acute phase while inducing persistent inflammation during remission (101). Stress-induced hyperactivity of the HPA axis leads to prolonged glucocorticoid elevation, which causes synaptic structural remodeling and disrupts negative feedback regulation - both of which are implicated in depression (102, 103). Notably, anxiety and stress can further exacerbate colitis by activating the HPA axis. In experimental models, dexamethasone (DEX) administration to simulate CORT secretion resulted in increased IL-6/TNF-α expression and significant downregulation of tight junction proteins occludin/ZO-1 (104). TNBS-induced colitis rats exhibited markedly elevated serum ACTH and CORT levels, though electroacupuncture (EA) treatment effectively alleviated both HPA axis hyperactivity and anxiety/depression-like behaviors (105). Collectively, these findings demonstrate intricate connections between the HPA axis, anxiety/depression, and inflammatory bowel diseases, though further research is warranted to elucidate the specific mechanisms underlying HPA axis involvement in IBD patients with comorbid psychiatric symptoms.

### 3.7 Tryptophan metabolic dysregulation

Recent research by Kennedy et al. confirms significant disruptions in tryptophan metabolism among IBD patients, characterized by an elevated kynurenine/tryptophan ratio. Under chronic inflammation, pro-inflammatory cytokines (e.g., IFN-γ, IL-6) induce indoleamine 2,3-dioxygenase (IDO) expression, redirecting approximately 95% of tryptophan toward the kynurenine pathway (106, 107). This depletes substrates essential for 5-HT synthesis.

The metabolic imbalance exerts dual detrimental effects: it directly reduces levels of neuroprotective brain-derived neurotrophic factor (BDNF), while concurrently enabling kynurenine metabolites to cross the BBB (108). These metabolites activate microglia and trigger neuroinflammation.

Notably, gut microbiota dysbiosis further disrupts tryptophan homeostasis (109), establishing a self-perpetuating vicious cycle of “gut inflammation - dysbiosis - neurotransmitter abnormalities”. This mechanistic framework provides novel insights into the high prevalence of mood disorders in IBD patients.

## 4 Emerging therapies for depression and anxiety in IBD

### 4.1 Microbial-gut-brain axis-targeted therapies

The orally administered hydrogel strategy (SP@Rh-gel) developed by Zhejiang University researchers co-delivers *Spirulina platensis* and rhein, significantly enhancing intestinal drug retention. This system inhibits the NF-κB-Caspase-1 inflammatory pathway, repairs the intestinal barrier, and reduces pro-inflammatory cytokines crossing into the brain. Preclinical studies confirm its dual efficacy in alleviating IBD symptoms and anxiety-depression behaviors by modulating the microbiome-gut-brain axis (MGBA) (110). SP@Rh-gel enhances drug solubility, controlled release, and intestinal retention, thereby improving oral bioavailability. It also rebalances disrupted gut microbiota and maintains intestinal barrier integrity, blocking pro-inflammatory cytokines (e.g., TNF-α, IL-6) and endotoxins (e.g., LPS) from entering the hippocampus via the BBB, thus suppressing neuroinflammation and preserving neural plasticity.

Recent clinical studies highlight specific bacterial strains (e.g., SCFA producers) as key regulators of gut-brain signaling. Restoring microbiota-derived metabolites (e.g., butyrate) enhances vagal neurotransmission and reduces central neuroinflammation (53, 111). German research identified inverse correlations between depression severity and abundances of SCFA-producing genera (e.g., *Odoribacter*, *Anaerostipes*) in IBD patients. Targeted supplementation with these bacteria modulates glycosaminoglycan (GAG) metabolic pathways, alleviating fatigue and depressive symptoms (80).

### 4.2 Neuromodulation technologies

Neuromodulation technologies provide innovative solutions for this clinical challenge by targeting the “gut-brain axis” (112). Among these, invasive vagus nerve stimulation (VNS)—approved by the U.S. FDA for treatment-resistant depression—involves implanting electrodes to directly stimulate cervical vagus nerves. This effectively regulates serotonergic neuronal activity in brain regions such as the locus coeruleus. A 2023 multicenter clinical study reported significant anxiety improvement in 58% of IBD patients receiving VNS, with therapeutic effects sustained over a 6-month follow-up period (113). While VNS offers stable neurotransmitter modulation, its surgical risks require careful evaluation.

In contrast, non-invasive VNS (nVNS) employs transcutaneous electrical stimulation to activate auricular (taVNS) or cervical (tcVNS) vagal branches. This technique delivers dual regulatory effects. Direct modulation of emotional circuits in the central nervous system. Improvement of intestinal inflammation via gut-brain-axis-mediated immunoregulation (114). Such dual neuromodulatory and immunomodulatory properties position nVNS as a potential therapy for IBD with comorbid refractory depression. However, current neuromodulation primarily targets depressive/anxiety symptoms, with limited clinical evidence specific to IBD populations (115).

### 4.3 Innovations in pharmacotherapy combination strategies

**Figure 2:**
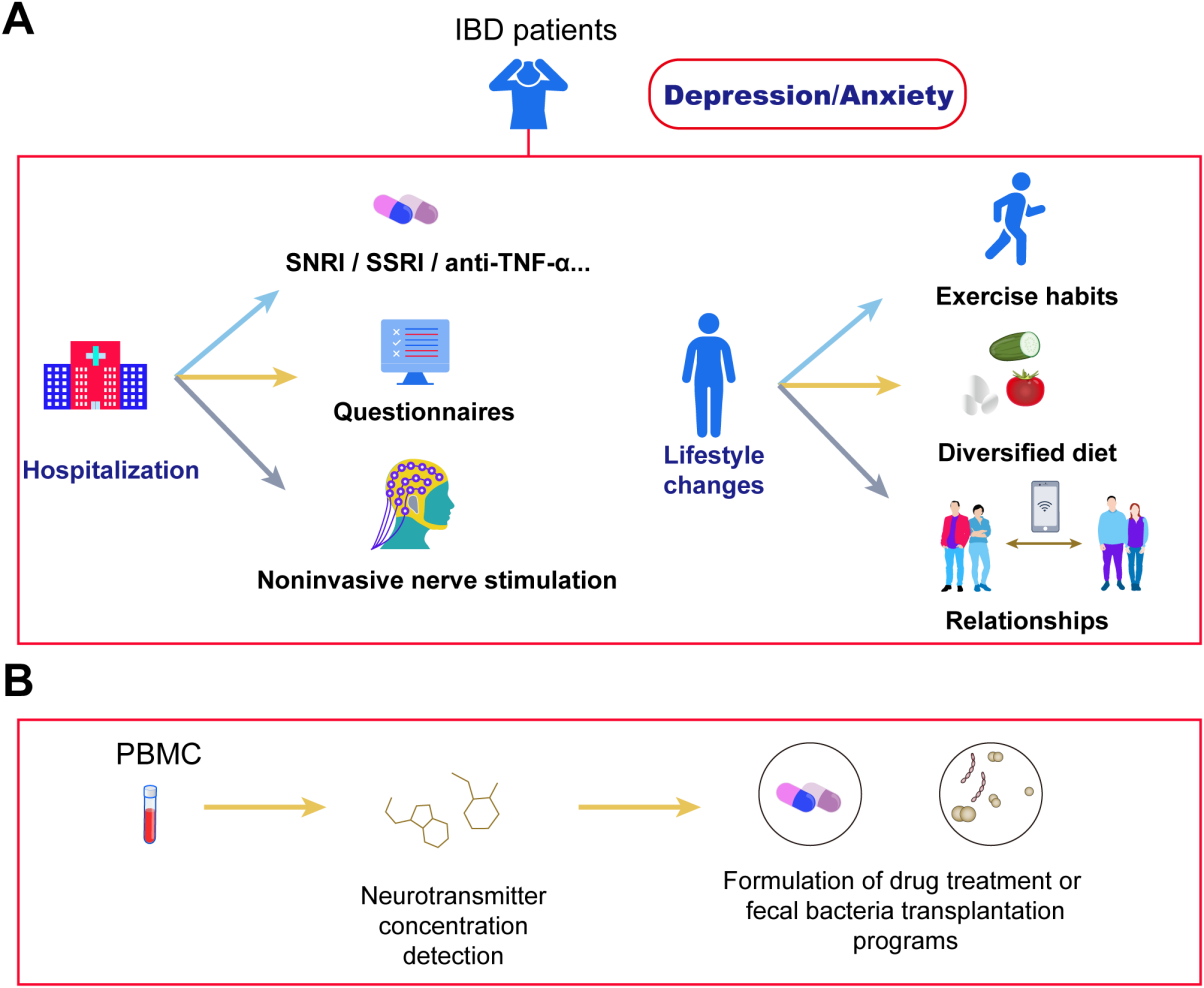
Cutting-edge therapies for depression and anxiety in IBD. **(A)** IBD patients with depression/anxiety can address symptoms through hospitalization or lifestyle adjustments. During hospitalization, symptoms improve via antidepressant/anxiolytic medications, questionnaire assessments, and non-invasive stimulation therapies. Lifestyle improvements include establishing regular exercise habits, adopting a diversified diet, and expanding social networks. **(B)** Blood neurotransmitter assays enable precise evaluation of depression/anxiety symptoms in IBD patients, facilitating targeted interventions. Examples include department-specific medication protocols to modulate neurotransmitter concentrations, and microbiota-targeted approaches (e.g., fecal microbiota transplantation or specialized formulations) that selectively modify gut microbiota structure to regulate neurotransmitter balance.

NE and 5-HT serve as critical neuroregulators in mood modulation for IBD patients. Human studies demonstrate significantly reduced colonic NE and 5-HT levels in the inflamed mucosa of both CD and UC patients compared to non-diseased controls (116, 117). The elevation of tissue NE/5-HT levels induced by SSRIs (selective 5-HT reuptake inhibitors) or SNRIs provides a mechanistic explanation for their observed protective effects in CD and UC management (4). Anti-inflammatory/antidepressant combination therapy synergistically alleviates mood symptoms through dual-pathway modulation1: Duloxetine (SNRI) combined with anti-TNF-α agents concurrently blocks peripheral inflammatory cytokines from crossing into the brain while inhibiting central monoamine reuptake, thereby achieving simultaneous intestinal mucosal healing and reduced Hamilton Depression Rating Scale (HAMD) scores (118); concurrently, microbial metabolite formulations such as butyrate sustained-release capsules activate intestinal epithelial FFAR receptors to enhance brain-derived neurotrophic factor (BDNF) expression, with Phase II clinical trials confirming their efficacy in alleviating depressive symptoms and reducing IL-1β levels, as illustrated in **Figure 2A**.

### 4.4 Complementary therapies

Various complementary therapies can synergize with primary interventions. Research indicates that anti-inflammatory diets and increased physical activity (PA) significantly alleviate anxiety and depressive symptoms in UC patients (119). Multiple studies confirm that higher anxiety/depression scores in IBD patients correlate strongly with sedentary behavior tendencies (120), while moderate-intensity leisure exercise improves psychological states (121); increasing activity levels (e.g., transitioning from moderate to high activity) reduces the CD activity index by an average of 25.3 points (122) (**Figure 2A**). Additionally, the high prevalence of anxiety and depression in IBD patients may deteriorate healthy eating beliefs into pathological pursuits of “pure” foods (123), with studies revealing a strong negative correlation between anxiety/depression and food-related quality of life (FR-QoL)—individuals with low FR-QoL exhibit reduced fiber intake (124, 125). Although animal experiments demonstrate anti-inflammatory effects from SCFAs produced by gut microbiota metabolism of dietary fiber, in IBD patients during active phases, frozen high-fiber foods may trigger gastrointestinal discomfort, and certain fiber types could exacerbate anxiety symptoms; thus, their efficacy for psychological symptom improvement requires further clinical validation.

Beyond dietary and exercise interventions, adjunct therapies like sleep regulation demonstrate potential value in IBD management despite limited current evidence (112, 115, 118). Monitoring peripheral blood neurotransmitter levels enables a three-tier prevention system: alterations in 5-HT and GABA levels facilitate early screening for psychological disorder risks (126); tracking dopamine dysfunction and acetylcholine/NE balance guides personalized treatments (127); and post-supplementation recovery of neurotransmitter levels (e.g., after SCFAs therapy) or enhanced 5-HT reuptake efficiency following SSRI administration serve as efficacy indicators (128). This framework offers novel insights for integrated management of comorbid psychological disorders in IBD (**Figure 2B**).

## 5 Summary and future directions

The review systematically delineates the epidemiological characteristics, pathological mechanisms, and clinical management strategies for anxiety and depression comorbidities in IBD patients. Research demonstrates that the prevalence of psychiatric disorders in IBD patients significantly exceeds that in the general population, with psychiatric symptoms and disease activity forming a vicious cycle. Genetic evidence indicates the first year post-IBD diagnosis represents the peak period for mental disorder risk. Pathological mechanisms involve: gut microbiota dysbiosis impairing neural function via reduced SCFAs and disrupted tryptophan metabolism; proinflammatory cytokines altering central nervous system activity through vagus nerve signaling; and systemic inflammation potentially compromising the blood-brain barrier and exacerbating neurological damage. Interventional approaches reveal that anti-inflammatory diets and exercise therapy significantly alleviate symptoms, while a neurotransmitter-monitored three-tier prevention system offers novel avenues for precision treatment.

This review has several key limitations that need to be addressed. Firstly, the clinical translation of mechanistic research remains inadequate, with targeted therapies such as microbial interventions and vagus nerve stimulation primarily confined to animal studies or small-scale clinical trials, lacking validation through large-scale randomized controlled trials (RCTs). Secondly, therapeutic strategies lack personalization, as multimodal interventions fail to provide stratified recommendations based on IBD subtypes, disease activity levels, or psychological symptom severity. Finally, there is insufficient interdisciplinary integration, with emerging advancements in behavioral psychology and digital health technologies not being adequately incorporated, creating a disconnect from the current “biopsychosocial” comprehensive treatment model. These gaps highlight the need for improved translational research, more rigorous methodologies, and better interdisciplinary collaboration in addressing IBD-related psychiatric comorbidities.

Future studies should focus on elucidating the mechanisms by which specific microbial strains regulate the gut-brain axis, developing microbiota-targeted dietary interventions and novel therapies such as vagus nerve stimulation, and optimizing personalized antidepressant regimens. Clinically, multidisciplinary collaboration should be enhanced to integrate mental health assessments into routine IBD care, thereby simultaneously improving both physical and psychological symptoms. These efforts will advance personalized and precision management of IBD comorbidities.
